# Estimating the Population of Unowned Free-Ranging Domestic Cats in Denmark Using a Combination of Questionnaires and GPS Tracking

**DOI:** 10.3390/ani12070920

**Published:** 2022-04-04

**Authors:** Helene Brøgger Nielsen, Helene Ane Jensen, Henrik Meilby, Søren Saxmose Nielsen, Peter Sandøe

**Affiliations:** 1 Department of Veterinary and Animal Sciences, University of Copenhagen, DK-1870 Frederiksberg C, Denmark; jks690@alumni.ku.dk (H.B.N.); scf887@alumni.ku.dk (H.A.J.); saxmose@sund.ku.dk (S.S.N.); 2 Department of Food and Resource Economics, University of Copenhagen, DK-1958 Frederiksberg C, Denmark; heme@ifro.ku.dk

**Keywords:** density, *Felis catus*, feral cats, GPS tracking, home range, population size, stray cats, unowned socialised cats, unsocialised cats

## Abstract

**Simple Summary:**

Unowned free-ranging domestic cats divide opinion. Some people object to them. They dislike the noise they cause and disapprove when they find their faeces in gardens and public places. Others are concerned about the cats’ welfare. It is widely believed that the number of unowned unsocialised cats (alleged to be 500,000) and additional unowned socialised cats in Denmark is huge. To assess whether this belief is correct, this study estimated the size of the population of unowned free-ranging domestic cats and their distribution in Denmark using a combination of questionnaires and GPS tracking. It was estimated that approximately 90,000 unowned cats are in the country, and that one-third are socialised and two-thirds unsocialised. It seems therefore that the number of free-ranging cats in Denmark that are unowned and unsocialised is only a fraction of that claimed, and that panic is unwarranted. The highest population density of unowned cats was found in rural areas.

**Abstract:**

The present study aimed (1) to estimate the size of the population of unowned free-ranging domestic cats in Denmark using a questionnaire survey combined with a GPS-tracking survey, and (2) to estimate the distribution of the population across different habitats. The questionnaires were circulated in 94 randomly selected parishes ranging across seven kinds of habitat. Using responses from five of the habitats, we estimated the population of unowned free-ranging cats nationally. In the other two habitats, questionnaire data were collected in a simpler way. The territory of 59 owned cats was estimated with GPS tracking to assess home ranges. Home range area was calculated using 95% Brownian bridge kernel density estimation (0.033–0.077 ± 0.011–0.023 km^2^, median ± SE). We estimated a population of unowned free-ranging cats in Denmark of 89,000 ± 11,000 (SE), with a mean density of 2 ± 0.3 (SE) cats per km^2^, living primarily in rural habitats. Approximately one-third of the cats were estimated to be socialised and two-thirds unsocialised. Our method may be suitable for use in other temperate areas facing problems with unowned free-ranging cats.

## 1. Introduction

Free-ranging domestic cats (*Felis catus*) can be found almost anywhere around the world where people reside [1,2]. Some have owners, but there are also many unowned animals, some of which are socialised (sometimes called ‘stray cats’) and some of which are unsocialised (sometimes called ‘feral cats’). Typically, the latter are not ‘feral’ in the biological sense, referring to semi-wild animals living independently of humans [3], as they often live close to humans on whom they depend to some extent for food.

Free-ranging cats divide opinion. Some worry that they negatively affect biodiversity and wildlife through their predation of other species [4,5,6,7] and transmit infectious agents to wild and domestic animals [8] and humans (e.g., rabies or toxoplasmosis [9,10,11]). In many places, including Denmark [12], they are also regarded by many people as a nuisance because they dig up soil, leave their faeces in gardens and public places, are noisy, and scare companion cats.

Others have concerns about the welfare of the cats. High mortality, increased rates of infection and disease, sometimes associated with feline immunodeficiency virus and feline leukaemia virus, vehicle trauma, starvation, the effects of harsh weather conditions, bite wounds and parasite infiltrations [3,13,14] are cited here. The authors of a recent paper warn that the ‘risk to biodiversity and public health’ presented by free-ranging cats should not be exaggerated as this may ‘fuel an unwarranted moral panic over cats’ [15].

The regulation of unowned free-ranging cats has led to various control strategies often involving collecting, trapping and killing. Increasing efforts have been made to introduce non-lethal methods to control populations. Typically, unowned socialised cats are taken into shelters—some are rehomed while others are euthanised. Some unsocialised cats are caught and, if healthy, neutered and released in trap–neuter–release (TNR) programmes, but in many places most of the trapped animals are euthanised [16].

In these respects, Denmark is unexceptional. Large numbers of unowned socialised cats are placed in Danish shelters owned and run by a range of animal welfare NGOs. In recent years, the rate has been over 10,000 cats per year. Most of these cats are rehomed [17]. Organised management of unsocialised cats is undertaken mainly by a single cat NGO, Kattens Værn. In the period 2009–2019, between 3000 and 5000 of these cats were caught and euthanised annually. As a result of the very restrictive Danish regulations covering the release of cats, only a smaller number of the unsocialised cats caught—between 400 and 800 per year—could go into a TNR programme (personal communication, Lone Nielsen, former CEO of Kattens Værn). A much smaller number were until recently caught and euthanised by a cat NGO working in and around the city of Aarhus, and an unknown number of unowned free-ranging cats were euthanised by veterinarians in private practice.

In Denmark, the ‘moral panic’ over unowned free-ranging cats can be seen in the strong opinions, often voiced, about their spread, invasive role in nature, influence on biodiversity and health conditions, and the potentially negative effects of feeding stations. This can also be observed in regulatory options using TNR strategies and legislative measures to improve their welfare [12]. In the public debate in Denmark, an estimate of 500,000 unsocialised cats—approximately the same number as that of owned cats in the country [18]—has circulated for a number of years. The origin of this figure seems to be a White Paper on animal welfare published by the responsible ministry in 1987 [19]. This stated that ‘there is no official information about the number of cats in Denmark. However, the committee [authoring the White Paper] estimates that there are around 500,000 unsocialised cats in towns and summer cottage areas.’ The committee added that these cats ‘are often sick and weak due to cold and lack of food’, thereby contributing to the sense of panic. A similar estimate was arrived at in a 1998 enquiry [20], but this study was based exclusively on information gathered in a small, localised countryside area in the middle of Region Zealand.

It can be seen therefore that in important respects, the situation in Denmark is presently unclear. Opinions on the numbers of unowned free-ranging cats, and on the corresponding need for interventions, are highly speculative. Thus, there is an urgent need to assess the size of the problem.

Population densities of free-ranging carnivores have been estimated using a range of methods: spotlight surveys of unsocialised cats [21], radio collar telemetry tracking of unsocialised cats [2,22,23,24,25], active track counts of coyotes [26], passive tracks of dingoes [7], scat counts of coyotes [27], live capture of bobcats [28], and landscape productivity as measured by satellite imagery [29]. The distribution of unsocialised cat populations in a selected habitat has been investigated in a number of countries and territories including the Galapagos Islands, Ecuador [2], Italy [30], Australia [21,23,31], South Africa [1], Sweden [32], Switzerland [33], and the USA [22,24,25,34]. Various habitats have been examined, most of which fall into three categories: rural [23], urban [24,25,35] and exurban [34].

The estimation of population size and the distribution of unowned free-ranging cats presents many challenges. First and foremost, discriminating between owned and unowned cats is challenging. Camera trapping may not separate them, as it can be difficult to determine whether a cat is unowned or an owned cat caught in the vicinity of the camera. Unsocialised cats are usually shy and difficult to catch [36]. Furthermore, several of the above-mentioned studies only estimate population density in a specific area, or habitat, and use a small sample size, often because of the major expense associated with studies such as these.

Given these methodological challenges, we decided to use questionnaire surveys to assist us in our estimation of the population size of unowned free-ranging cats and their distribution in Denmark. Thus, local respondents’ knowledge of the cats and their potential origin was used as a basis for assessing the number of cats in a certain area. This approach has been used at a much smaller scale in two studies from the USA [22] and Denmark [20], and it was recently used in a national-scale study conducted in the United Kingdom (UK) [37]. What is new in our study is that we randomly selected areas in each kind of cat habitat and then scaled up the results using information about habitat area and the size of cat territories to arrive at an estimate of the population of unowned free-ranging cats in the country as a whole.

The objectives of this study were: (1) to estimate the population size of unowned free-ranging cats using a questionnaire survey in combination with a GPS-tracking survey designed to determine the home ranges of the cats; (2) to estimate the distribution of unowned free-ranging cats in different habitats; and (3) to examine the validity of this new approach to estimating the population size and distribution of unowned free-ranging cats in a large geographical area.

## 2. Materials and Methods

### 2.1. Outline of Method Used and the Assumptions on Which It Is Based

We divided Denmark into seven different cat habitats. For five of these, the rural, suburban, summer cottage, urban, and industrial habitats, we used a questionnaire survey of people living in randomly selected parishes to assess the number of unowned free-ranging cats in each specific habitat. It was then possible to extrapolate from the resulting estimates to an estimate of the population size of unowned free-ranging cats nationally. Thus, information at the micro level on the selected habitats was upscaled to the macro level using national statistics on the area coverage of specific habitats and estimates of home range areas, or territory sizes, based on a GPS study. In this way, we arrived at an estimate of the size of the total population of unowned free-ranging cats and their distribution. For a further description of sampling and estimation procedures and an exemplification of the calculations performed, see Appendix C.

The two remaining types of habitats were investigated as follows. For harbour habitats, we sent questionnaires to the relevant people; and for areas with no human residence or commercial use, we used telephone interviews.

This method presupposes that in densely populated temperate countries such as Denmark, unowned free-ranging cats live near humans. Table 1 presents an overview of the extent, in km^2^, of the habitats and the different types of land use in Denmark.

In this study, we focus on areas where people live and/or work: harbours, rural areas (by which we mean the intensively used parts with buildings and gardens), suburban areas, summer cottage areas, urban areas, and industrial areas. In addition to these areas, Denmark has three other kinds of areas: (1) a mixed bag of natural habitat and areas used by humans (lakes, basins, runways, raw material areas, wetland, forest, scrub vegetation, moor and sand dunes) labelled ‘no human residence’ in Table 1, (2) areas with agriculture and horticulture, and (3) residuals, e.g., roads and railways. We assessed the presence of unowned free-ranging cats in (1) in consultation with those managing what seemed to be a likely cat habitat, namely the forests. We presumed that cats in (2) are all seen and counted in the habitat described as ‘rural areas’. Finally, we assumed that no unowned free-ranging cats would be present in (3). Note that rural habitats include areas located around villages, farms, and isolated houses, and are therefore fragmented, and that this leads to some uncertainty about the boundaries of the habitat and area used by the cats.

We further assumed that people are familiar with the area in which they live and can therefore provide reasonably accurate estimates of the number of cats in their local vicinity. The general assumption that unowned free-ranging cats live close to humans is supported by previous studies. These have concluded that in areas with high food availability, cat densities are high, at >50 individuals/km^2^ [40]; and that in areas with low food resources, the cat density is lower, at 5–50 individuals/km^2^ [41]. Unsocialised cats will, of course, try to remain at a safe distance from people, but the assumption is that they still live close to humans, mainly feed on anthropogenic food sources, and will therefore be seen by the humans around them. The assumption that unsocialised cats have anthropogenic diets is supported by a Danish study of the stomach contents of cats found killed on the road, which concluded that nearly 80% of the content during winter and spring, and 60% during summer and fall, was commercial cat food [42].

A further assumption is that the home ranges of owned socialised cats, which were tracked as part of this study, will be similar to those of unowned free-ranging cats. This assumed similarity finds some support in the research literature [25]. However, there is also anecdotal evidence that unsocialised cats have larger home ranges.

A final assumption is that cats can be placed neatly into the categories ‘owned’, ‘unowned and socialised’ and ‘unowned and unsocialised’. This is of course a simplification. Unsocialised cats are shy and fearful of human interaction [43], whereas unowned socialised cats may have had an owner previously or some other form of human contact early in life and are therefore used to humans. They can be tame and confident in situations of cat–human interaction [3]. A possible middle group would be cats that are not admitted to households but live in present or former farm buildings—so-called ‘farm cats’. These may be shy and may not be fully recognised, or acknowledged, as owned cats. However, we assumed that people would classify them as owned.

### 2.2. GPS-Tracking Study

#### 2.2.1. GPS-Tracking Study Design

GPS data from owned free-ranging cats over one year old were collected by H.A.J. with the help of veterinary students of year 2021 attending a research integration project at the University of Copenhagen. Owners and their cats were recruited through social media. To collect data on the cats’ home range, 20 Tractive GPS devices (Tractive GmbH, Pasching, Austria) were used. Each device weighed 28 g, was waterproof and shock resistant, and had between 2 and 5 days of battery life [44]. The error margin of the GPS coordinates was reported as approximately 7.8 m, and coordinates were calculated by triangulation of mobile phone towers. The GPS was fitted either with a safety collar or with a collar with buckle clasps, depending on whether the cats persistently removed the former. Data on the sex, age and neuter status of the cats were collected from their owners (Appendix B).

#### 2.2.2. GPS-Tracking Data Collection

GPS data from participating cats were collected between 30 July 2021 and 6 November 2021. Locations were recorded every 2–60 min, depending on the activity level of the cat. Fixes were less frequent if cats were inactive or indoors, and more frequent if owners activated live-tracking mode. The cats were tracked for 6–7 days. Cats not already accustomed to wearing a collar had an adaptation period of 1–5 days prior to data collection. The cat’s owner evaluated whether it had accepted the collar, and cats refusing to wear a collar were excluded from this study.

#### 2.2.3. GPS-Tracking Data Management and Analysis

Geographical coordinates collected by the GPS receivers were downloaded and saved as GPX files. With the help of the Garmin Basecamp software package for Windows, the GPX files were converted and exported as CSV files for analysis in R.

Home range areas were estimated using Brownian bridge kernel density estimation. In this way, any spatial dependence between locations visited by the cats was taken into account. Smoothing parameters were estimated for each individual cat using maximum likelihood estimation, and home range areas representing 95% of the utilisation distribution were calculated (here abbreviated BBKDE 95%). The utilisation distributions were estimated using the kernelbb function in the adehabitatHR package in R [45]. The median, mean and standard deviation of home range areas were estimated for cats from suburban, rural and summer cottage habitats.

### 2.3. Questionnaire Study: Outline

The size of the population of unowned free-ranging cats and their distribution were estimated with six different questionnaires collecting primary data on the observed number of cats of different types in different habitats (see below) together with GPS tracking results giving estimates of the home range areas of the cats. Three different methods were used to estimate the number of unowned free-ranging cats in the different habitats:

(1) In suburban areas, summer cottage areas and rural areas (which included low-density housing and industrial areas), we calculated the mean number of cats across respondents. Next, the total population size of unowned free-ranging cats in an area category was estimated by multiplying the average number of cats reported with the habitat area divided by the median home range size (see more details on how to do this in Appendix C).
(2) In harbour areas, the mean number of unowned free-ranging cats was estimated using the same method as in (1), but the estimate of the total size of the population across harbours was based on the mean number in each of the three harbour types (described below in the section Questionnaire Study: Design) multiplied by the number of harbours of this type.
(3) In industrial and urban areas, the mean density of unowned free-ranging cats was estimated by dividing the total number of cats reported by the area covered by the respondents’ properties. Next, the total size of the population of unowned free-ranging cats in these areas was estimated as the mean density of cats multiplied by the total habitat area.

In all habitat types, population size was summarised as a total, and then in each of the two sub-categories of unowned cats, i.e., socialised and unsocialised.

To summarise, (1) uses habitat area and home range area; (2) uses the total number of harbours but not home range area or the area of the habitat; and (3) uses habitat area and the area of respondents’ properties but not home range area. The methods are elaborated below in the section Questionnaire Study: Data Analysis. Different approaches are used in the different habitats for feasibility reasons. In the suburban areas, summer cottage areas and rural areas we investigated, it was possible to survey randomly selected inhabitants with a well-defined geographical spread. Where the harbours were concerned, we could actually send the survey to the person, or persons, managing each of the harbours in the country. In the industrial and urban areas, it was not possible to randomly select areas. We simply had to try to elicit answers from any person we could contact who was managing the relevant industrial facility, apartment block and so on.

The study period was April 2020–June 2021. The mean temperature in Denmark in 2020 was 9.8 °C, the highest temperature being 32.4 °C and the lowest being −8.2 °C [46]. Annual precipitation was 773.0 mm. The Danish (human) population in 2020 was 5,822,763 [47] and the total area of Denmark is 42,947 km^2^ [39]. The topography of the country is generally flat. The country is mostly surrounded by the sea, and the highest point is 173 m above sea level.

### 2.4. Questionnaire Study: Habitats and Study Period

**Figure 1:**
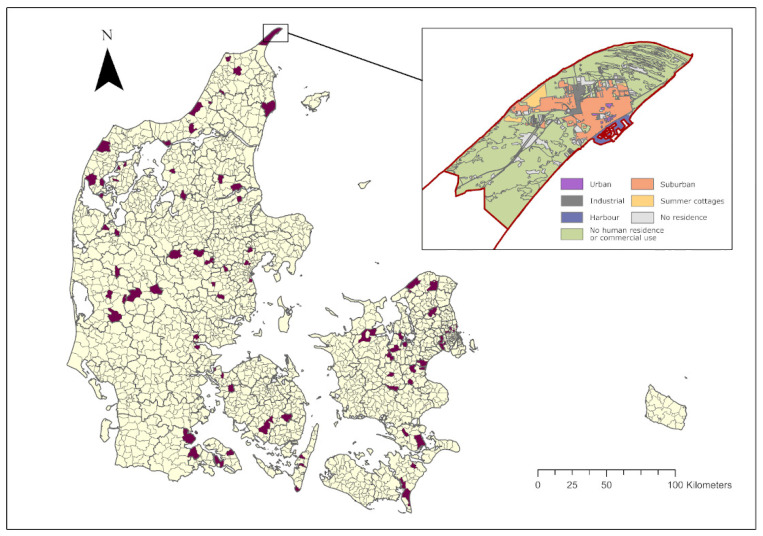
Sampled areas in Denmark (purple)—32 municipalities with 3 parishes in each. Skagen Parish is shown with habitat types. Based on map data provided by The Danish Agency for Data Supply and Efficiency, Copenhagen NV, Denmark, and Danish Municipalities; ‘DAGI’, ‘GeoDanmark’, August 2020 [38].

In the habitats rural areas, urban areas, industrial areas, summer cottage areas and suburban areas, the questionnaire survey was only conducted in selected sample areas. In the five regions of Denmark, 32 municipalities (out of a total of 98) with 3 parishes in each municipality were randomly selected using SAS (v.9.4, Cary, NC, USA) to take part in the survey. The sampled parishes are shown in Figure 1. One of the thirty two municipalities (Glostrup) had only one parish, but it provided a quantity of data similar to that obtained in the other municipalities with three parishes. The selected areas are listed in Appendix A.

To describe the areas covered by each habitat type in more detail, Denmark was divided into three zones: (1) the rural zone, (2) the summer cottage zone and (3) the urban zone, with (3) being further divided into four different types of use: (i) city centre, (ii) low-density housing, (iii) high-density housing and (iv) industrial areas. To apply these distinctions, we used maps provided by SDFE (The Danish Agency for Data Supply and Efficiency, Copenhagen NV, Denmark), an agency holding a public database with information about the Danish geosystem [38].

Denmark was further divided into the NUTS 2 regions: the Capital Region, Region Zealand, the Region of Southern Denmark, the Central Region of Denmark and the Northern Region of Denmark. NUTS (nomenclature of territorial units for statistics) is a hierarchical system for dividing the economic territory of the EU and the UK. The remote island Bornholm, which is part of the Capital Region, was only considered in relation to the harbour habitat, because we decided to collect data from islands forming part of Denmark only if they have a bridge connection with the mainland. Some properties extended across both sides of a border between two regions. These were divided into two polygons, divided by the border, in order to calculate the total area of each habitat in each region.

The habitat types considered to be of relevance and therefore included in this study were either inhabited areas or intensively used areas around buildings. The only exception to this was the habitat type ‘no human residence’. The total area of Denmark and the areas of the selected habitats are shown in Table 1.

### 2.5. Questionnaire Study: Design

#### 2.5.1. Harbour

The questionnaire distributed to harbour masters checked that the respondent represented a harbour and it was designed to: (1) provide information about the respondent including gender and age, along with the name of the harbour represented, (2) describe the type of harbour represented, (3) state whether the respondent had observed any cats at the harbour within the last month and, if so, how many, (4) record whether the cats the respondent had observed were believed to be unowned and free-ranging, and (5) specify how many of these cats were tame (and therefore belonged to the group of unowned socialised cats) and how many were shy (and were therefore likely to be unsocialised).

#### 2.5.2. Rural Area, Summer Cottage Area and Suburban Area

Overall, the questionnaires used in these three habitats were designed to provide a partial validation of the responses received. This was achieved by asking respondents how many different (1) unowned, (2) unowned and tame, or (3) unowned and shy cats they had observed, (a) *on their property,* and (b) within a radius of *200 m outside their property* within (i) *one week,* and (ii) *one month*. Hence, 12 different responses were received from each respondent. The reason for asking about (1) the total number of observed unowned cats, and then asking, ‘*out of those unowned cats observed, how many would you describe as (2) tame and (3) shy?’*, was to validate whether (1) the total number of unowned cats matched the sum of (2) unowned socialised and (3) unsocialised cats. Information about the study respondents including age, gender and occupational status was collected together with their addresses. Informed consent was also obtained.

#### 2.5.3. Industrial and Urban Area

The questionnaire applied in industrial and urban areas was designed to (1) validate whether the respondent represented a company or had an affiliation with a housing complex, (2) provide information about the respondent, and (3) describe number of cats seen, as for the rural, urban and summer cottage areas.

### 2.6. Questionnaire Study: Data Collection

The questionnaires developed for the different habitat types were tested in a pilot study run in the town of Bogense, located on Funen, in the Region of Southern Denmark on 10 June 2020. Bogense was selected for the pilot because it represented all the habitats used in this study. The pilot revealed that several adjustments to the questionnaires were required. The questions were simplified, with fewer response options, and the order in which the questions were asked was changed.

#### 2.6.1. Harbour Habitat

Questionnaires were e-mailed to all 356 harbour masters in Denmark on 25 May 2020 using available contact information [48]. The survey period was from 25 May 2020 to 8 October 2020. A reminder was sent on 24 June 2020.

#### 2.6.2. Residential, Summer Cottage and Rural Habitats

The questionnaire surveys of the rural areas, summer cottage areas and suburban areas were carried out in person with the assistance of 112 first-year Veterinary Bachelor of Science Students at the University of Copenhagen as a part of a research integration project taking place during a first-year course on ‘Veterinary ethics and philosophy of science’ (2020), for which the senior author of this paper was responsible. The students, who worked in groups of 3–4, collected the data by ringing on doorbells and filling out the questionnaires on site. In suburban areas, the students were instructed to make contact with residents in every 5th house on the road. In rural areas and summer cottage areas, every house on the road was visited. The period of the survey was 3–4 October 2020. The students were instructed on how to perform the questionnaire survey in a joint meeting nine days prior to the survey. They were also provided with written instructions supplying them with information about their sampling area.

#### 2.6.3. Industrial and Urban Habitat

The questionnaires for the industrial areas and urban areas were sent out by e-mail between 17 December 2021 and 16 March 2021. Reminders were sent on 7 and 18 February and 8 March.

In the 32 municipalities selected for this study, there were 137 housing associations, found using Google maps. Of these, 76 had made an e-mail address publicly available. In the industrial area habitat, 200 industrial corporations were identified. E-mail addresses were publicly available for 150 of these, and after eliminating duplicates 148 questionnaires were sent out. In both the urban and industrial areas, the addresses from which responses had been obtained were looked up in the SDFE map, and the cadastre numbers were identified and the property areas noted and summed.

#### 2.6.4. No Human Residence Habitat

To obtain information on the habitat type ‘no human residence’, 18 forest wardens were contacted. Details of all of the forest wardens were gathered from a webpage [49] and the wardens were contacted for an informal telephone interview on 27 May 2020 and again on 28 June 2020.

### 2.7. Questionnaire Study: Data Management

#### Habitats

Following compilation of all the data from the questionnaires, the data were checked for duplicates, errors in addresses and incomplete answers. Address errors were identified using ArcGis (ArcMap Software. Redlands, CA, USA, Esri Inc.) with the kind assistance of Assistant Professor Lene Jung Kjær, who matched the addresses with existing addresses. Data management was conducted using R (R Core Team).

The statistical software package SAS (v. 9.4, SAS Institute Inc.) was used to handle the databases. Estimators for stratified sampling were used to estimate variances and standard errors, and to estimate population size in each stratum and at the national level. National data on the distribution in an area of different habitats [39] were used to upscale to the country level.

### 2.8. Questionnaire Study: Data Analysis

#### 2.8.1. Harbours

We divided the 356 harbours in Denmark into three types: marina, commercial fishing harbour, and industry/ferry port. Information confirming harbour type was obtained from an official webpage providing information about all harbours in Denmark [39]. Marinas were characterised as harbours with sailing boats and small fishing boats. Commercial fishing harbours could also include small sailing boats, but they were characterised as having commercial fishing boats. Industry/ferry ports included harbours with an active industry and/or ferry terminal but usually also small sailing boats.

Total population of unowned free-ranging cats in the harbour habitat was estimated as the sum of the products of: (a) the mean number of (i) unowned cats, (ii) unowned socialised cats, or (iii) unsocialised cats, as observed by respondents representing a specific type of harbour, and (b) the share of this specific type of harbour, and (c) the total number of harbours. Means and standard errors were calculated using standard approaches to stratified sampling.

#### 2.8.2. Rural, Summer Cottage and Suburban Areas

To estimate the size of the population of unowned free-ranging cats and their distribution, we multiplied the mean number of (1) unowned cats, (2) unowned socialised cats, and (3) unsocialised cats by the total area of the habitat divided by the median home range of the cats. Standard error for the total size of the population of unowned free-ranging cats in these areas was calculated using the sum of the squared coefficients of variation of (i) the mean value of the number of unowned free-ranging cats and (ii) the median home range (see more on this in Appendix C).

Population density in each habitat was calculated as the estimated number of cats in the habitat divided by the habitat area for the total group of unowned free-ranging cats.

#### 2.8.3. Urban and Industrial Areas

Mean densities of cats for the three categories (1) unowned cats, (2) unowned socialised cats and (3) unsocialised cats were calculated by dividing the total number of cats reported by the respondents by the total area of the respondents’ properties. The mean density of unowned free-ranging cats was then multiplied by the total area of the urban or industrial habitat to estimate the population size here. Given the small sample size, no standard error was calculated.

#### 2.8.4. Population Density in Denmark

Total population density in Denmark was calculated as the total number of unowned free-ranging cats divided by the total area of Denmark. Uncertainty associated with the density estimate was based on the standard error of cat numbers divided by the total area of Denmark. Population density in the selected habitats was calculated as for the population density in Denmark.

## 3. Results

### 3.1. GPS Tracking

A total of 59 owned free-ranging cats contributed GPS data. Of those, 29 were females (one intact and 28 neutered) and 30 were males (two intact and 28 neutered). They ranged from 1 to 16 years of age (Appendix B). The cats were tracked for seven days, except for two which were tracked for six days. Of the 59 cats, 7 lived in a summer cottage area, 20 lived in a rural area and 32 lived in a suburban area.

The estimated mean, median and standard deviation of the home range areas were, respectively, 0.053, 0.033 and 0.051 km^2^ in suburban areas, 0.082, 0.077 and 0.036 km^2^ in summer cottage areas and 0.086, 0.057 and 0.081 km^2^ in rural areas. Within our data, the area of home ranges varied from a minimum of 0.010 km^2^ (suburban) to a maximum of 0.368 km^2^ (rural).

### 3.2. Estimation of Population Density and Distribution in Each Habitat

#### 3.2.1. Harbour

A total of 88 out of 356 harbours participated in the survey. The distribution of the three harbour types, their total numbers and the resulting sample size for each harbour type are shown in Table 2. The total size of the population of unowned free-ranging cats was estimated at 250 ± 66 (SE) cats (shown in Table 3). 

#### 3.2.2. Rural, Summer Cottage and Suburban Areas

The students collecting data from suburban, summer cottage and rural areas contacted a total of 3798 households, of which 1879 participated in the survey, giving an overall response rate of 49% (Table 4).

#### 3.2.3. Industrial and Urban Areas

Only a small number of responses were obtained from the industrial and urban areas. Therefore, some regions were not represented in the dataset. Of the 76 urban housing complexes we identified, only 28 participated (37%) and completed the questionnaire. In industrial areas, the total number of respondents was 24 (16%) of the 148 invited. The sampled area covered 0.8 km^2^ (0.2%) out of 441 km^2^ in the selected industrial area and 0.79 km^2^ (0.6%) out of 141 km^2^ in the selected urban area. Due to the lack of data, no standard errors were calculated for the population estimates. Results from the urban areas and industrial areas are listed in Table 5, which also shows the estimated number of unowned free-ranging cats in each habitat.

#### 3.2.4. No Human Residence Areas

Just over half (10) of the 18 forest wardens who were contacted participated in interviews. None believed they had seen cats that looked as if they were unowned and free-ranging in the forest they managed. The eight wardens not participating did not answer their telephones. On this basis, we assumed that there were no unowned socialised or unsocialised cats in this habitat.

### 3.3. Total Estimation of Population Density and Distribution

Based on the BBKDE 95% for home range, we obtained national totals of approx. 89,000 ± 11,000 (SE) unowned, 23,000 ± 4000 unowned socialised, and 44,000 ± 7000 unsocialised domestic cats (Table 5). We conjecture that the reason why the two last numbers do not add up to 89,000 is that, except in industrial and urban areas, people were asked separate questions about these categories of cats and, in some cases, were unsure how to categorise some of the unowned cats they saw.

The total population density of unowned free-ranging cats in the selected habitats in Denmark was estimated at 6 ± 1 cats per km^2^ (mean ± SE) based on the BBKDE 95% (Table 6). The population density of unowned free-ranging cats in the total area of Denmark was 2 ± 0.3 cats per km^2^. The rural habitat category had the highest density of cats.

## 4. Discussion

Using a combination of a questionnaire survey for different habitats and GPS tracking to estimate cats’ home range areas in some of the habitat types, we estimated that there are in total approx. 89,000 ± 11,000 (SE) unowned free-ranging domestic cats in Denmark. Approximately one-third of these were estimated to be socialised and two-thirds unsocialised. If our estimate is accurate, the widely cited figure of 500,000 unsocialised cats in Denmark, first published by the responsible ministry in 1987 [19], is approximately eight times too large. This result may, perhaps, serve to counteract any actual or potential ‘moral panic’ over the prevalence of unowned cats in Denmark. This said, there are, of course, good reasons to continue efforts to promote neutering, marking and registering of owned cats in order to avoid an increase in and even to limit the number of unowned cats.

Other studies have estimated the population density of unowned free-ranging cats in an area comparable in size to Denmark. In Illinois, USA, an estimate of free-ranging farm cats in a rural habitat was based on the mean number of farm cats on a rural property multiplied by the total number of rural properties in the state of Illinois, providing a result of 4.0 ± 1.8 (SE) million rural cats [22]. The same method, using the mean number of cats multiplied by the total number of properties, was used in a now out-of-date (and unpublished) Danish study which suggested that 511,196 owned rural cats were in existence in 1998 [20]. The study did not include any estimates of uncertainty and was based on a single rural area of 47 km^2^ in Denmark. Neither of these studies factored in the home range area of the cats. A third and more recent study from the Netherlands estimated that there were between 135,590 and 1,207,331 unowned, or ‘stray’, cats in the Netherlands [50]. Finally, a recent study from the UK found a total population of 247,429 (95% credible interval: 157,153 to 365,793) unowned cats in the country [37]. Unlike our study, the UK study did not separate socialised and unsocialised unowned cats.

Looking at the UK study, we think it makes most sense to compare our results with those from England, which is the part of the UK most like Denmark in terms of its urbanisation. The UK study estimates a total of 193,698 unowned cats in England. Since the area of England is approximately three times that of Denmark, the Danish population of unowned cats should therefore be approximately 65,000, assuming the same population density in the two countries. This is approximately 73% of what we found. We think this disparity is due to limitations of the UK study. Most importantly, the areas it investigated were not randomly selected across the country and the data were from urban areas only. The authors write that “whilst we provide the first estimate of unowned cats in the UK, we are only able to account for urban areas that make up approximately 13% of total land cover. With the exception of cats on farms, that have access to increased resources, densities of unowned cats in rural areas are anticipated to be much lower…” (p. 6). In fact, the authors assume there are very few cats in rural areas in their calculations. Our findings indicate that this assumption is unsafe, as we found a higher density of unowned cats in the area around farm buildings than in any of the other areas surveyed. On this basis, we suggest that the numbers in the UK study are probably a significant underestimate.

The methodological problems and uncertainties in previous studies (not the UK study, which was published after our study had concluded) led us to use a method combining a questionnaire with GPS tracking to estimate home range area. We believe that this method could be used in other countries and regions where there are concerns about the size of the unowned free-ranging cat population.

### 4.1. GPS Study—Discussion of Limitations in Light of the Literature

The calculation of the median home range area of free-ranging cats was carried out with information on 59 owned free-ranging cats that were monitored for 6–7 days. To obtain more accurate estimates of mean home range, longer-term monitoring would be useful, as would seasonal monitoring. Short snapshots of home range add uncertainty. However, the monitoring period was chosen because it corresponded to the period for which respondents were asked to provide information.

Other GPS-tracking studies report varying home range estimates of unowned free-ranging cats. In Illinois, USA, the average home range area of both male and female free-ranging farm cats in an urban area was estimated at 155 ± 40 ha, corresponding to 1.55 ± 0.4 km^2^ [22]. On the Galapagos Islands, Ecuador, the mean home range area of feral cats on two islands without human settlement with the same vegetation and habitat was recorded as 3.04 km^2^ for males and 0.82 km^2^ for females [2]. In Australia, in a semi-arid woodland environment, the long-term estimate of mean home range of male feral cats was 2210.5 ha (22.10 km^2^); but over a shorter 24 h term, the mean home range was much smaller, at 250 ha (2.5 km^2^) [23]. In South Africa, in a campus locality situated in an urban conservation area, the mean home range of kittens, juvenile and adult feral cats combined ranged between approx. 4 and 11 ha, corresponding to 0.04–0.11 km^2^ [51]. In Caldwell Texas, in an suburban setting, the mean annual home range area for feral cats using a 50% kernel was 1.4 ha (0.001 km^2^), while it was 10.4 ha (0.01 km^2^) when a 95% kernel was used [24]. In Russellville, Arkansas, in an ex-urban area, the home range of feral cats was male 29.1 ± 7.7 ha (0.29 ± 0.07 km^2^) and female 12.26 ± 2.9 ha (0.12 ± 0.029 km^2^) [34]. Finally, in a rural area in Revninge, Sweden, a study of home range areas differentiated between sex, type of cat (feral or domestic) and, where the males were concerned, measures of social rank. Feral females had a home range of 206 ± 31 ha (2.06 ± 0.31 km^2^) and the home range of feral male challengers was 298 ± 11 ha (2.98 ± 0.11 km^2^) [52]. In comparison, the home ranges from our GPS-tracking survey were smaller, with mean home ranges of 0.053 km^2^ (median 0.033) in the suburban areas, 0.082 km^2^ (median 0.077) in the summer cottage areas and 0.086 km^2^ (median 0.057) in the rural areas. Factors that may explain this disparity will now be considered.

In addition to human population density, climate could have an impact on home ranges of the cats. It is reasonable to assume that cats living under the same climatic conditions and with similar human population density and infrastructure will have similar home range patterns. The considerable differences in the home ranges reported in existing studies could additionally be connected with differences in food supply and neuter status of the cats [24,25,30,31,35,52,53,54], or with the body weight of the cats, or access to shelter. However, food distribution appears to be the most important variable [53].

The studies mentioned above focus on the home ranges of free-ranging cats of different sorts, such as free-ranging farm cats [3], whereas our study was limited to owned free-ranging cats. Even though both categories of cats appear to be largely reliant on anthropogenic resources, this could have affected our estimates of home range, and thus our estimates of the size of the population of unowned free-ranging cats and their distribution in Denmark.

### 4.2. Questionnaires, Home Ranges and Habitats—Discussion of Limitations in Light of the Literature

#### 4.2.1. Questionnaires

This study used six different questionnaires. Those used in suburban, summer cottage, industrial, urban, and rural areas were comprehensive and asked about the number of unowned free-ranging cats in 12 different ways. The harbour questionnaires were less complex and only included three different ways of asking about the number of unowned free-ranging cats. The high number of ways of asking could have affected the results by confusing the respondents with too many similar questions. However, it also provided a partial validation of the answers. Of course, the way a survey is conducted can introduce various kinds of bias linked to human observation, and bias can also inform the observations made by researchers.

#### 4.2.2. Habitats

Other studies only investigated densities, or home range areas, in one habitat in a specific locality or region, e.g., highly urbanised areas [24,35,51], wilderness habitats [21] and rural properties [20,22]. Estimates of density in one habitat do not translate easily into estimates for a larger geographical area such as a country. In this study, seven habitats were selected to reflect the habitats found in Denmark. This selection made it possible to investigate the comparative distribution of the cats. It was then confirmed that the habitat with the highest density of unowned free-ranging cats was rural areas, which contained 26 ± 6 cats (mean ± SE) per km^2^.

The limited sample size where urban and industrial areas were concerned may have led to underestimation of the number of unowned free-ranging cats in these habitats. Additionally, the estimate for these areas was based on limited data, with the consequence that no standard errors could be calculated. Some of the industrial addresses were found in areas other than the industrial area identified in the SDFE map, giving rise to another uncertainty. Hence, our estimate of the number of unowned free-ranging cats in these areas is less well confirmed. However, since these are areas where there seem to be very few cats, we doubt that this had a significant effect on our overall estimate of the size of the population of unowned free-ranging cats in Denmark.

For the habitat type ‘no human residence’, it was estimated from the interviews with forest wardens that the number of cats was zero. The forest wardens were the only people available to take questions about this habitat, but their knowledge only covers, and they were only asked about, the forests. The rest of this habitat, including lakes, basins, runways, raw material area, wetlands, scrub vegetation, moors, and sand dunes, was not covered, and this may have biased the resulting estimate. However, other studies suggest that areas with low food availability have low cat densities [51,53], so the impact on our overall estimate is likely to be low.

In this study, the main cat habitats were divided into three zones, (1) urban, (2) summer cottage and (3) rural, covering different areas characterised as low-density housing, high-density housing, city centre, urban areas, industrial areas. The distribution of the zones, and the areas they covered, was determined by each municipality, and there was therefore no single, general definition of what the zones included. This could also have affected the results, as the habitat areas played an important role in our calculation of cat numbers.

### 4.3. Overview of Uncertainties and Limitations of This Study and the Method Applied

In light of the above, and the assumptions presented in Section 2.1, we see the following as the main limitations of our study:

First, there is uncertainty about the assumption that most, if not all, unowned free-ranging cats in a temperate, densely populated country such as Denmark live where there are people. Given our findings for forests, and looking at the literature, we feel reasonably confident that this assumption can be safely made. However, unowned free-ranging cats may roam where there are no people. If this is the case, our estimate is too low.

Second, there are uncertainties linked to the assumption that people actually are able to spot the the unowned free-ranging cats living in the area in which they themselves live and that they don’t mistake some owned cats for being unowned. This is the assumption we feel least confident about.

Our lack of confidence is supported by the recent UK study [37] mentioned above, which compares the number of cats recorded in surveys with expert assessments made on a number of sites and concludes that “across surveys, there was 17.92% (15.63 to 20.5; 95% CRI) probability of detecting an unowned cat when present and on average 0.72 (0.67 to 0.77) owned cats were misidentified as unowned at each site.” So, following this, we should both adjust our numbers upwards to account for underreporting of unowned cats and adjust them downwards to account for reported unowned cats which are in reality owned. However, when we consider the evidence presented for these numbers, we are not too impressed. The expert data only appear to be representative of the citizen data (i.e., overlap area wise) in two or three of the study areas, and they are not compared with a random sample of the citizen data. Given this, it is not possible to verify the accuracy of the correction factors, and we are reluctant to use these as that would merely add another bias. Furthermore, while correction for the detection probability is straightforward, our study includes no natural equivalent of the ‘sites’ used in the UK study, and thus to correct for the number of owned cats mistakenly reported as unowned cats (by subtracting 0.72 per site), we would need to arbitrarily introduce such ‘sites’, possibly leading to additional bias.

So, considering the recent UK study there are both reasons for us to think that our estimate of unowned free-ranging cats is too low because people don’t see all unowned free-ranging cats in their neighbourhood and too high because they mistake some owned cats for being unowned. Our guess it that the first factor is the most important and that our estimate therefore probably is too low.

Third, there are uncertainties linked to the assumption that our measures of home ranges for owned cats are comparable to what would be found if the home ranges of unowned free-ranging cats were measured. The literature suggests that the home ranges of unowned free-ranging cats are not dramatically larger than those of owned cats, but it may be that some unowned free-ranging cats, notably intact males, have considerably larger home ranges than those we found. If they do, our estimate may be too high.

Fourth, there are uncertainties linked to the assumption that the habitat areas defined by the applied map dataset are representative of the areas actually used by cats. Particularly with the rural habitat category, this can be doubted, as this habitat type includes intensively used areas (gardens and courtyards) around villages, farms and scattered buildings. This habitat type is in fact highly fragmented, with an extensive perimeter, and cats may use a larger or smaller area than that represented by the formal habitat category. Some rural buildings are uninhabited, which could imply that our estimate is too high. On the other hand, gardens around isolated houses are often fairly small, which could mean that cats also use surrounding parts of the landscape and that our estimate is thus too low. We believe that the latter effect would dominate the former, and thus that, if anything, our estimate is too low.

In short, one uncertainty points in the direction of overestimation and three in the direction of underestimation. Given that it is the second assumption about which we are least confident, with underestimation as a consequence, our considered view is that the most likely form of inaccuracy in this study is that our estimate of Danish cat numbers is too low. We will now assess this possibility by comparing an alternative approach suggested in the literature.

### 4.4. Comparison with an Alternative Approach

In a meta-study, Bengsen et al. [29] remodelled the data collated by Liberg et al. [53], who had prepared regression models relating home range size and density. Males and females were modelled separately, and the mean squared errors (MSE) were estimated at 0.314 for females and 0.668 for males. The mean home range size of male cats generally exceeded that of females in the datasets used by Liberg et al. [53]. In our data, the mean home range size of males (M) was larger than that of females (F) in rural areas (M 12.1 vs. F 5.8 ha). In suburban areas, mean home range sizes were similar for males and females (M 5.4 vs. F 5.2 ha); and in summer cottage areas, the mean home range size of males was seemingly smaller than that of females (M 7.1 vs. F 9.6 ha). The sample size was, however, also small in summer cottage areas (M: *n* = 4, F: *n*= 3). Using our estimates of density in suburban, rural and summer cottage areas (Table 6), and predicting the log home range size of female cats, the prediction errors were all negative but in no case lower than −1.96 RMSE.

Inverting the model used by Liberg et al. [53] for female cats (ln(range size, ha) = 5.0875–0.7988 ln(density, km^2^)), and using habitat areas from Table 1, the estimated number of cats would be 111,107 in suburban areas, 10,279 in summer cottage areas and 110,158 in rural areas. The total would then be 231,544, i.e., almost three times our estimate (34,682 + 44,522 + 3120 = 82,324) (Table 5 and Table 6). Assuming even shares of male and female cats, and using overall mean densities for the three habitats (suburban: 5.3 ha, summer cottages: 8.2 ha, and rural: 8.6 ha) together with models for male (50%) and female (50%) cats, an estimate of 185,925 + 21,703 + 115,960 = 323,588 results. Given the small mean home range estimates for male cats, even larger numbers would result applying the model Liberg et al. [53] use to males cats.

The small mean home ranges that we observed for male cats could be a consequence of our having sampled owned free-ranging cats, most of which are neutered (Appendix B). However, given the control programmes implemented in Denmark, it is likely that a share of unowned free-ranging cats are neutered, implying that male range size in Denmark may be lower than predicted by the Liberg et al. model.

The large difference between our population estimates and those obtained using the Liberg et al. model can partly be explained by the uncertainties described above, including the possibility that respondents are not aware of all of the unowned cats that live on, or cross, their land. However, there are also at least three reasons why these models are likely to overestimate the population of unowned free-ranging cats in Denmark.

First, Refs. [29,53] draw on a variety of studies from different parts of the world, some of which focus on populations of cats that are not in contact with humans, while others concentrate on cats living with and around humans. Liberg et al. [53] noted that part of the wide scatter around their regression lines was caused by cases of cats that obtain food from their owners. A higher correlation was achieved when such studies were omitted, and Bengsen et al. [29] deliberately excluded studies conducted around human settlements. By contrast, our study emphasises populated rural and urban habitats, where a considerable share of the resident free-ranging cats is owned by people, and unlike the model-based estimates our population estimates represent only the unowned free-ranging segment of the overall population. This segment is likely to be small in many cases. For example, Liberg [41] found that only 10% of cats in a rural study area in Skåne (Southern Sweden) were unowned. While owned cats living in human households fall outside the scope of our questionnaire survey, they nevertheless interact with unowned free-ranging cats within their home range. They can therefore be expected to affect the density of unowned free-ranging cats negatively. For a given home range size, it therefore appears unlikely that the density of unowned free-ranging cats will be as high as it would have been if the area were not to a considerable extent ‘saturated’ by owned cats.

Second, as also mentioned by Liberg et al. [53], a factor that seriously affects population density is human control. In Denmark, the populations of unowned free-ranging cats are, as mentioned above, regulated. This regulation presumably helps to reduce the population density of such cats, taking it below the densities predicted by the regression models of Liberg et al. [53], irrespective of home range size.

Third, home range studies vary considerably in their sampling procedures, duration of tracking period and methods used to calculate home range size. Bengsen et al. [29] excluded studies where cats were tracked for less than 15 days to avoid cases where cats might not be able to reveal the full extent of their home range. In our study, the cats were tracked for only 6–7 days. For some cats, this could imply that the estimated mean home range sizes are slightly underestimated relative to the values used in calibration of the Liberg et al. [53] models, thus leading to overestimates of density and population size when these models are used.

In sum, comparison with the model-based approach suggests that our estimate of Danish cat numbers is too low. That said, there are good reasons to believe that the application of the model-based approach delivers a result that is too high.

## 5. Conclusions

This study provides an estimate of the size of the population of unowned free-ranging cats and their distribution in Denmark. The estimated population size was approx. 89,000 ± 11,000 (SE) cats, corresponding to a density in the selected habitats of 6 ± 1 (SE) cats per km^2^ based on the BBKDE 95% home range and 2.1 ± 0.3 (SE) cats in the total area of Denmark. Of these cats, two-thirds are unsocialised and one-third are socialised.

The distribution of the cats across the selected habitats showed that the habitat with the highest density of cats was rural areas, which had 26 ± 6 (SE) cats per km^2^.

With the provisos mentioned above, the method used in this study seems to have been effective in delivering an estimate of the size of the population of unowned free-ranging cats and their distribution in Denmark. Of course, a degree of uncertainty remains. We have drawn attention to four sources of it, one of which suggests that our estimate is too high, and three of which suggest, by contrast, that it is too low.

Our main methodological concern is that people may not always see the unowned free-ranging cats at large in the area in which they live. That would imply that our estimate is too low. This worry is reinforced when an alternative model-based approach is compared with ours. However, we continue to think that our estimate is a reasonably accurate indication of the size of the problem of unowned free-ranging cats in Denmark, and that the figure of 500,000 widely cited is a significant exaggeration.

Our results indicate that in Denmark, the ‘problem’ of unowned cats, to the extent that there is one, is not out of control. Therefore, there seems to be no need for increased trapping and euthanasia of unowned unsocialised cats outside certain local areas where there are particular concerns either about vulnerable populations of wild animals or rapid growth in the local unowned cat population.

## Figures and Tables

**Table 1:** Habitats and land use in Denmark in km^2^. The habitat type ‘no human residence or commercial use’ includes lakes, basins, runways, raw material areas, wetland, forest, scrub vegetation, moor and sand dunes.

Habitat Type	Area (km^2^)
Harbour	-
Rural ^†^	1700
Suburban ^†^	1509
Summer cottage ^†^	298
Urban ^†^	141
Industrial ^†^	441
No human residence ^†^	9696
Total (habitats) ^†^	13,785
Agriculture and horticulture *	25,787
Residuals, e.g., roads and railways *	3375
Total (Denmark) ^†^	42,947

^†^ Based on map data provided by the Danish Agency for Data Supply and Efficiency [38]. * Based on Statistics Denmark [39].

**Table 2:** Overview of the harbours in Denmark, total number, number sampled and the share of each type.

Harbour Type	Sampled (*n*)	Total in DK (*n*)	Share
Marina	63	309	0.87
Commercial fishing	21	26	0.07
Industrial/ferry port	4	21	0.06
Total	88	356	1

**Table 3:** Estimated population of unowned free-ranging cats in the Danish harbours, and standard error (SE, shown in brackets).

	Unowned Cats	Unowned Socialised Cats	Unsocialised Cats	Unowned Socialised + Unsocialised Cats
Total Number	250	182	182	364
Standard Error	(±66)	(±102)	(±58)	(±161)

**Table 4:** Participant distribution. This table shows the number of participants in the habitats (% in brackets).

Area Type	Participated	Not Home	Did Not Want to Participate	Total
Rural	679 (52)	502 (38)	125 (10)	1306 (100)
Summer Cottage	129 (28)	290 (62)	48 (10)	467 (100)
Suburban	1071 (53)	669 (33)	285 (14)	2025 (100)
Total	1879 (49)	1461 (39)	458 (12)	3798 (100)

**Table 5:** Total populations in Denmark. Estimated population size of unowned free-ranging domestic cats from questionnaire survey data and BBKDE 95% home ranges by habitat type and in total. Standard errors are given in brackets.

Habitat	Unowned Cats	Unowned Socialised Cats	Unsocialised Cats
Suburban Areas	34,682 (±5763)	6187 (±1469)	9704 (±2097)
Rural Areas	44,522 (±9618)	14,214 (±3342)	26,877 (±6486)
Summer Cottage Areas	3120 (±698)	776 (±260)	1974 (±646)
Harbours	250 (±66)	182 (±102)	182 (±58)
Industrial Areas ^†^	6040	1098	4942
Urban Areas ^†^	820	285	535
No Human Residence or Commercial Use Areas	0	0	0
Total	89,433 (±11,234)	22,743 (±3662)	44,214 (±6847)

^†^ For industrial and urban areas, the number of unowned cats is estimated as the sum of socialised and unsocialised cats.

**Table 6:** Total population numbers in Denmark. Estimates of unowned free-ranging cat population sizes by habitat type and for Denmark as a whole.

Area Type	Area (km^2^)	Home Range	Number of Cats				Cat Density Per km^2^			
			Estimate	SE ^1^	95% CI ^1^		Mean	SE	95% CI	
Rural	1700	BBKDE 95%	44,522	9618	25,286	63,758	26	6	15	38
Suburban	1509	BBKDE 95%	34,682	5763	23,157	46,207	23	4	15	31
Summer cottage	298	BBKDE 95%	3120	698	1724	4515	10	2	6	15
Urban	141	NA	820	-			6			
Industrial	441	NA	6040	-			14			
No human residence or commercial use	9696	NA	0	-						
Harbour	NA	NA	250	66	118	382				
Total (habitats)	13,785	BBKDE 95%	89,433	11,234	66,965	111,901	6	1	5	8
Total (area of Denmark)	42,947	BBKDE 95%	89,433	11,234	66,965	111,901	2	0	2	3

^1^ SE = standard error; 95% CI = 95% confidence interval. BBKDE 95% = 95th percent of the utilisation distribution obtained using Brownian bridge kernel density estimation.

## Data Availability

All data from the study, apart from those presented in Appendix A, Appendix B, Appendix C are available in the Supplementary Materials S1-file.

## Supplementary Materials

The following is available online at https://www.mdpi.com/article/10.3390/ani12070920/s1. Supplementary Materials S1. Areas and questionnaire data.xlsx.

## Appendix A

**Table A1:** List of the parishes in the five regions with 32 municipalities with 3 parishes randomly selected for this study (Glostrup only had one parish).

Parish	Municipality	Region Name	Parish Code	Municipality Code	Region Code
Engholm	Allerød	Capital Region	9155	201	1084
Lillerød	Allerød	Capital Region	7450	201	1084
Lynge	Allerød	Capital Region	7425	201	1084
Brøndby Strand	Brøndby	Capital Region	9084	153	1084
Brøndbyøster	Brøndby	Capital Region	7145	153	1084
Brøndbyvester	Brøndby	Capital Region	7144	153	1084
Helleruplund	Gentofte	Capital Region	7110	157	1084
Skovshoved	Gentofte	Capital Region	7116	157	1084
Vangede	Gentofte	Capital Region	7113	157	1084
Haralds	Gladsaxe	Capital Region	9071	159	1084
Mørkhøj	Gladsaxe	Capital Region	7134	159	1084
Søborgmagle	Gladsaxe	Capital Region	9068	159	1084
Glostrup	Glostrup	Capital Region	7147	161	1084
Esbønderup	Gribskov	Capital Region	7428	270	1084
Tibirke	Gribskov	Capital Region	7394	270	1084
Vejby	Gribskov	Capital Region	7393	270	1084
Lisbjerg	Aarhus	Central Region Denmark	8127	751	1082
Mejlby	Aarhus	Central Region Denmark	8248	751	1082
Skåde	Aarhus	Central Region Denmark	8104	751	1082
Assing	Herning	Central Region Denmark	8809	657	1082
Kølkær	Herning	Central Region Denmark	9119	657	1082
Tiphede	Herning	Central Region Denmark	9205	657	1082
Ødum	Ringkøbing-Skjern	Central Region Denmark	8785	760	1082
Sædding	Ringkøbing-Skjern	Central Region Denmark	8776	760	1082
Troldhede	Ringkøbing-Skjern	Central Region Denmark	9124	760	1082
Gjern	Silkeborg	Central Region Denmark	8027	740	1082
Grønbæk	Silkeborg	Central Region Denmark	8596	740	1082
Thorning	Silkeborg	Central Region Denmark	8601	740	1082
Ry	Skanderborg	Central Region Denmark	9104	746	1082
Sjelle	Skanderborg	Central Region Denmark	8075	746	1082
Tåning	Skanderborg	Central Region Denmark	8040	746	1082
Fousing	Struer	Central Region Denmark	8824	671	1082
Hjerm	Struer	Central Region Denmark	8820	671	1082
Lyngs	Struer	Central Region Denmark	8656	671	1082
Albæk-Lyngså	Frederikshavn	Northern Region Denmark	8447	813	1081
Hulsig	Frederikshavn	Northern Region Denmark	9228	813	1081
Skagen	Frederikshavn	Northern Region Denmark	8480	813	1081
Astrup	Hjørring	Northern Region Denmark	8460	860	1081
Em	Hjørring	Northern Region Denmark	8415	860	1081
Horne	Hjørring	Northern Region Denmark	8463	860	1081
Arden	Mariagerfjord	Northern Region Denmark	9184	846	1081
Sem	Mariagerfjord	Northern Region Denmark	8203	846	1081
Vive-Hadsund	Mariagerfjord	Northern Region Denmark	9191	846	1081
Bjergby	Morsø	Northern Region Denmark	8672	773	1081
Rakkeby	Morsø	Northern Region Denmark	8699	773	1081
Skallerup	Morsø	Northern Region Denmark	8678	773	1081
Heltborg	Thisted	Northern Region Denmark	8686	787	1081
Vang	Thisted	Northern Region Denmark	8730	787	1081
Vestervig	Thisted	Northern Region Denmark	8648	787	1081
Aggersborg	Vesthimmerlands	Northern Region Denmark	8399	820	1081
Fjelsø	Vesthimmerlands	Northern Region Denmark	8520	820	1081
Hyllebjerg	Vesthimmerlands	Northern Region Denmark	8353	820	1081
Brovst	Jammerbugt	Northern Region Denmark	8393	849	1081
Alstrup	Jammerbugt	Northern Region Denmark	8423	849	1081
Hune	Jammerbugt	Northern Region Denmark	8425	849	1081
Maglebrænde	Guldborgsund	Zealand Region	7608	376	1085
Nykøbing F	Guldborgsund	Zealand Region	7578	376	1085
Væggerløse	Guldborgsund	Zealand Region	7580	376	1085
Hagested	Holbæk	Zealand Region	7294	316	1085
Kundby	Holbæk	Zealand Region	7296	316	1085
Soderup	Holbæk	Zealand Region	7286	316	1085
Ålsemagle	Køge	Zealand Region	7207	259	1085
Ejby	Køge	Zealand Region	7216	259	1085
Vollerslev	Køge	Zealand Region	7538	259	1085
Herslev	Lejre	Zealand Region	7163	350	1085
Kirke Såby	Lejre	Zealand Region	7175	350	1085
Sæby	Lejre	Zealand Region	7181	350	1085
Havdrup	Solrød	Zealand Region	7202	269	1085
Jersie	Solrød	Zealand Region	7204	269	1085
Solrød	Solrød	Zealand Region	7203	269	1085
Kalvehave	Vordingborg	Zealand Region	7481	390	1085
Mern	Vordingborg	Zealand Region	7480	390	1085
Udby	Vordingborg	Zealand Region	7477	390	1085
Benløse	Ringsted	Zealand Region	7364	329	1085
Sneslev	Ringsted	Zealand Region	7376	329	1085
Vetterslev	Ringsted	Zealand Region	7373	329	1085
Felsted	Aabenraa	Southern Region Denmark	9023	580	1083
Genner	Aabenraa	Southern Region Denmark	9260	580	1083
Løjt	Aabenraa	Southern Region Denmark	9012	580	1083
Brahetrolleborg	Faaborg-Midtfyn	Southern Region Denmark	7746	430	1083
Espe	Faaborg-Midtfyn	Southern Region Denmark	7750	430	1083
Gislev	Faaborg-Midtfyn	Southern Region Denmark	7768	430	1083
Bagenkop	Langeland	Southern Region Denmark	9254	482	1083
Fuglsbølle	Langeland	Southern Region Denmark	7707	482	1083
Simmerbølle	Langeland	Southern Region Denmark	7742	482	1083
Gelsted	Middelfart	Southern Region Denmark	7832	410	1083
Roerslev	Middelfart	Southern Region Denmark	7828	410	1083
Strib	Middelfart	Southern Region Denmark	7826	410	1083
Notmark	Sønderborg	Southern Region Denmark	8993	540	1083
Sankt Marie	Sønderborg	Southern Region Denmark	8988	540	1083
Ulkebøl	Sønderborg	Southern Region Denmark	8992	540	1083
Hornstrup	Vejle	Southern Region Denmark	7896	630	1083
Vinding	Vejle	Southern Region Denmark	7917	630	1083
Vor Frelsers	Vejle	Southern Region Denmark	7894	630	1083

## Appendix B

**Table A2:** Information about the cats involved in the GPS study on home range area.

Cat	Sex	Age (Years)	Neutered	Area	Municipality	Region
1	Male	7	Yes	Suburban	Gribskov	Capital Region of Denmark
2	Female	1	Yes	Suburban	Gribskov	Capital Region of Denmark
3	Male	2	Yes	Summer cottage	Gribskov	Capital Region of Denmark
4	Male	8	Yes	Summer cottage	Gribskov	Capital Region of Denmark
5	Male	3	Yes	Summer cottage	Gribskov	Capital Region of Denmark
6	Female	4	Yes	Summer cottage	Gribskov	Capital Region of Denmark
7	Female	3	Yes	Summer cottage	Gribskov	Capital Region of Denmark
8	Female	3	Yes	Summer cottage	Gribskov	Capital Region of Denmark
9	Female	2	Yes	Rural	Gribskov	Capital Region of Denmark
10	Male	9	Yes	Rural	Stevns	Region Zealand
11	Female	3	Yes	Rural	Gribskov	Capital Region of Denmark
12	Male	8	Yes	Summer cottage	Gribskov	Capital Region of Denmark
13	Male	2	No	Rural	Furesø	Capital Region of Denmark
14	Female	4	Yes	Rural	Thisted	Region of Northern Denmark
15	Female	12	Yes	Rural	Furesø	Capital Region of Denmark
16	Female	12	Yes	Suburban	Horsens	Central Denmark Region
17	Male	10	Yes	Rural	Halsnæs	Capital Region of Denmark
18	Male	4	Yes	Rural	Sønderborg	Region of Southern Denmark
19	Male	1	No	Rural	Viborg	Central Denmark Region
20	Female	2	Yes	Rural	Næstved	Region Zealand
21	Male	13	Yes	Suburban	Albertslund	Capital Region of Denmark
22	Male	5	Yes	Suburban	Esbjerg	Region of Southern Denmark
23	Female	4	Yes	Suburban	Ballerup	Capital Region of Denmark
24	Male	11	Yes	Suburban	Fredensborg	Capital Region of Denmark
25	Male	10	Yes	Rural	Roskilde	Region Zealand
26	Female	10	Yes	Suburban	Ballerup	Capital Region of Denmark
27	Male	1	Yes	Rural	Faxe	Region Zealand
28	Male	8	Yes	Suburban	Rudersdal	Capital Region of Denmark
29	Female	16	Yes	Suburban	Rudersdal	Capital Region of Denmark
30	Male	9	Yes	Suburban	Aarhus	Central Denmark Region
31	Male	10	Yes	Rural	Gribskov	Capital Region of Denmark
32	Male	12	Yes	Suburban	Kolding	Region of Southern Denmark
33	Female	5	Yes	Rural	Slagelse	Region Zealand
34	Male	4	Yes	Rural	Tappernøje	Region Zealand
35	Female	1	No	Suburban	Egedal	Capital Region of Denmark
36	Male	9	Yes	Suburban	Gribskov	Capital Region of Denmark
37	Male	6	Yes	Suburban	Køge	Region Zealand
38	Female	2	Yes	Suburban	Roskilde	Region Zealand
39	Male	3	Yes	Suburban	Faxe	Region Zealand
40	Male	15	Yes	Suburban	Gentofte	Capital Region of Denmark
41	Female	4	Yes	Rural	Aabenraa	Region of Southern Denmark
42	Female	4	Yes	Rural	Aabenraa	Region of Southern Denmark
43	Male	3	Yes	Suburban	Herning	Central Denmark Region
44	Male	2	Yes	Suburban	Herning	Central Denmark Region
45	Female	4	Yes	Suburban	Herning	Central Denmark Region
46	Male	3	Yes	Suburban	Slagelse	Region Zealand
47	Male	1	Yes	Suburban	Slagelse	Region Zealand
48	Female	9	Yes	Suburban	Guldborgsund	Region Zealand
49	Female	5	Yes	Suburban	Brønderslev	Region of Northern Denmark
50	Female	2	Yes	Suburban	Brønderslev	Region of Northern Denmark
51	Female	12	Yes	Rural	Sønderborg	Region of Southern Denmark
52	Female	7	Yes	Rural	Sønderborg	Region of Southern Denmark
53	Female	8	Yes	Rural	Sønderborg	Region of Southern Denmark
54	Female	9	Yes	Suburban	København	Capital Region of Denmark
55	Female	3	Yes	Suburban	København	Capital Region of Denmark
56	Male	3	Yes	Suburban	København	Capital Region of Denmark
57	Male	1	Yes	Suburban	Lolland	Region Zealand
58	Female	1	Yes	Suburban	Lolland	Region Zealand
59	Female	10	Yes	Suburban	Aarhus	Central Denmark Region

Study on home range of owned free-ranging cats collected by H.A.J. in 2021.

## Appendix C. Sampling and Estimation Procedures

The density of unowned free-ranging cats was expected to vary across habitats. In addition, some regional variation was expected, and we therefore decided to sample each of Denmark’s five regions separately. Within each region a random sample of households would have been ideal, but since we planned to conduct interviews on site a totally random sample was deemed impractical. We therefore applied a multi-stage procedure instead.

First, we sampled 6–7 random municipalities within each region. Second, we sampled 3 randomly selected parishes within each municipality (one municipality, however, only included a single parish). Third, roads and streets passing through areas classified as suburban, rural and summer cottage areas (habitat types) were selected, and households were visited and interviewed by teams of students. In suburban areas, every fifth house on a road was visited. In rural and summer cottage areas, all of the houses on a road were visited. Each team of students worked for two days and was responsible for conducting interviews in one municipality. Not all habitat types occurred in all parishes or municipalities, and we therefore estimated the mean number of unowned free-ranging cats observed on properties within the last week at the level of habitat type within region.

The GPS-tracking data were collected using an alternative approach. Here, owners of free-ranging domestic cats were recruited partly through social media and partly with the assistance of veterinary Bachelor’s students. This enabled us to identify a total of 59 cats within suburban, rural and summer cottage areas. However, in this dataset, the only region in which all three habitat types were represented was the Capital Region (around Copenhagen), and we therefore analysed home range size at the level of habitat type for the country as a whole.

Based on maps provided by The Danish Agency for Data Supply and Efficiency [37] we calculated the area of each of the three habitat types by region.

Based on the household interviews, we estimated the mean number of observed unowned free-ranging, unowned socialised and unowned unsocialised cats for each habitat type in each region. We assigned equal weights to all respondents.

Using 95% Brownian Bridge Kernel Density estimates of home range, we estimated mean and median home range size for each habitat type. Sample sizes were only 7–32 cats per habitat type, and since the distribution of home range size was also skewed, we used median home range size in our analysis.

We assumed that the mean number of unowned cats reported by respondents in a given habitat type and region (${\bar{n}}_{h,r}$) was directly proportional to the density of cats ($d_{h,r}$) in that area. We also assumed that density and home range size (${\hat{a}}_{h}$) were inversely proportional. Hence,

$$d_{h,r}=\frac{N_{h,r}}{A_{h,r}}=k\frac{{\bar{n}}_{h,r}}{{\hat{a}}_{h}},\;$$

where k is a coefficient, $N_{h,r}$ is the size of the cat population and $A_{h,r}$ is the area of habitat h∈[1;3] in region r∈[1;5].

Reorganising the equation and assuming k=1, the number of cats can be estimated as

$${\hat{N}}_{h,r}={\bar{n}}_{h,r}\frac{A_{h,r}}{{\hat{a}}_{h}},\;$$

where the ratio $\frac{A_{h,r}}{{\hat{a}}_{h}}$ specifies the number of cats the area would hypothetically host if the whole area was used and no territories overlapped, implying that all parts of the area would be used by exactly one cat. The mean number of cats observed, ${\bar{n}}_{h,r}$, acts as a coefficient specifying the number of cats using an area monitored by a respondent. Provided that (1) this area is smaller than the typical size of territories, (2) cats are evenly distributed within the habitat, (3) on average, respondents observe and are able to distinguish between cats using the area within the one-week recall period, and (4) the estimated home range size is representative of unowned free-ranging cats, we would expect this estimator to be unbiased. As described in the Discussion section, there are, however, reasons to believe that our estimates may be too low.

We estimated the total number of cats within each habitat type ($N_{h}$) by summing up the results across regions. Hence,

$${\hat{N}}_{h}=\overset{5}{\underset{r=1}{\sum }}{\hat{N}}_{h,r}=\overset{5}{\underset{r=1}{\sum }}{\bar{n}}_{h,r}\frac{A_{h,r}}{{\hat{a}}_{h}}\;$$

We will now look at an example. For the suburban habitat type, the average number of unowned cats observed by respondents in the five regions and the corresponding areas of the habitat are shown below in Table A3. The median home range estimated for this habitat type was 3.2514 ha. Using these results, the estimated number of cats in the suburban habitat was

$${\hat{N}}_{h}=0.63\frac{28935}{3.25}+0.68\frac{38396}{3.25}+0.84\frac{20621}{3.25}+0.88\frac{24349}{3.25}+0.77\frac{38617}{3.25}=34682$$

This value is included in Table 5 and Table 6.

When calculating the standard error of the estimates, we assumed that habitat area was without uncertainty. Hence, for each region, the variance of the estimate could be estimated as the sum of the squared coefficient of variation of the mean number of cats observed, $c^{2}\left({\bar{n}}_{h,r}\right)$, and the squared coefficient of variation of the median home range size, $c^{2}\left({\hat{a}}_{h}\right)$, multiplied by the squared estimate of the number of cats, ${\hat{N}}_{h,r}^{2}$, i.e.,

$$s^{2}\left({\hat{N}}_{h,r}\right)={\hat{N}}_{h,r}^{2}\times \left(c^{2}\left({\bar{n}}_{h,r}\right)+c^{2}\left({\hat{a}}_{h}\right)\right)\;$$

Finally, the standard error of the total number of cats within a habitat was calculated from the sum of the variances estimated for the five regions, i.e.,

$$s\left({\hat{N}}_{h}\right)=\sqrt{\overset{5}{\underset{r=1}{\sum }}s^{2}\left({\hat{N}}_{h,r}\right)}\;$$

**Table A3:** Average number of cats observed by respondents together with the areas (ha) of the suburban habitat within each of the five regions in Denmark are shown below.

	Capital Region	Central Jutland	North Jutland	Zealand	Southern Denmark
Average no. of Cats Observed	0.634	0.681	0.842	0.875	0.766
Habitat area (ha)	28,935	38,396	20,621	24,349	38,617
